# Role of Male Factor Testing in Recurrent Pregnancy Loss or *In Vitro* Fertilization Failure

**DOI:** 10.4172/2161-038X.1000e122

**Published:** 2015

**Authors:** Ryan Dickey, Ranjith Ramasamy

**Affiliations:** 1 Department of Urology, Baylor College of Medicine, Houston, Texas, USA; 2 Department of Urology, University of Miami – Miller School of Medicine, Miami, Florida, USA

## Editorial

The appropriate management for male partners of couples with recurrent pregnancy loss (RPL) or recurrent implantation failure during *in vitro* fertilization (IVF) remains unclear. In particular, men with normal semen parameters are often ignored because the “bulk semen parameters” appear normal [1]. Despite normal semen parameters, male partners in couples with RPL or recurrent implantation failure could have underlying genetic abnormalities in sperm DNA that can be identified. There are a couple of diagnostic tests that we recommend in the evaluation of these men, the first being DNA Fragmentation Index (DFI) and the second, fluorescence in situ hybridization (FISH) for evaluating sperm aneuploidy.

DNA fragmentation occurs due to abnormal packaging of sperm chromatin. Unlike somatic cells that utilize histones as the primary packaging of chromatin, sperm utilize protamines. First, approximately 85% of histones are replaced by protamines, tightly winding chromatin into structures called toroids [2]. In the epididymis, protamines are further compacted by disulfide bond cross-linking. Sperm becomes susceptible to damage if packing with protamines is incomplete. In particular, there are no repair mechanisms that occur once sperm are transported to the epididymis or post ejaculation [3]. High DNA damage as demonstrated by increased DFI is associated with recurrent pregnancy loss, recurrent IVF failure, and increased congenital abnormalities [4,5]. Therefore, men with abnormally elevated DFI can undergo testicular biopsy for sperm retrieval and use with intracytoplasmic sperm injection (ICSI) because DFI in testicular sperm is significantly lower compared to DFI in ejaculated sperm [6]. There are several DFI assays available, and each has its own set of advantages and disadvantages. The sperm chromatin structure assay (SCSA) is commercially available, but the Terminal deoxynucleotidyl transferase (TdT) dUTP Nick-End Labeling (TUNEL) assay, Halo test, and Comet assay are other options.

The second test that can be utilized in men with RPL is sperm aneuploidy testing. Sperm aneuploidy is defined as any deviation from the normal haploid state of sperm. Because many chromosomal aberrations are not viable, clinical testing of sperm aneuploidy has centered on those compatible with survival, namely trisomy 13, 18, 21, X monosomy and Klinefelter (XXY-XXXXY) [7]. The main cause of aneuploidy is nondisjunction, but anaphase lag and ineffective checkpoint control can also contribute to abnormal numbers of chromosomes. Sperm FISH is a cytogenic assay that measures the frequency of chromosomal abnormalities by measuring sperm aneuploidy. FISH can be effective in identifying abnormal sperm aneuploidy in men with RPL and normal sperm parameters. Up to 40% of men with RPL and normal semen parameters had abnormal aneuploidy with FISH [8,9]. Our recommendation for men with increased sperm aneuploidy is to pursue IVF with preimplantation genetic screening (PGS) or early fetal DNA testing in maternal blood.

Taken together, both DFI and FISH testing are recommended in the work-up of male factor in couples with recurrent pregnancy loss or recurrent IVF failure. Men with increased DFI in ejaculated sperm may be counseled for a testicular biopsy in combination with ICSI, and those with increased sperm aneuploidy can be advised to undergo IVF combined with PGS.
